# What do people write about COVID-19 and teaching, publicly? Insulators and threats to newly habituated and institutionalized practices for instruction

**DOI:** 10.1371/journal.pone.0276511

**Published:** 2022-11-10

**Authors:** Mario Antonio Martinez

**Affiliations:** College of Education, The University of Houston, Houston, Texas, United States of America; King Khalid University, SAUDI ARABIA

## Abstract

Covid represents major changes in teaching across the world. This study examined some of those changes through tweets that contained threats and insulators to habitualization of newer teaching practices. The investigator harvested tweets to determine sentiment differences between teaching and schools and teaching and online. Topic modeling explored the topics in two separate corpora. Omnibus Yuen’s robust bootstrapped t-tests tested for sentiment differences between the two corpora based on emotions such as fear, anger, disgust, etc. Qualitative responses voiced ideas of insulation and threats to teaching modalities institutionalized during the pandemic. The investigator found that ‘teaching and school’ was associated with higher anger, distrust, and negative emotions than ‘teaching and online’ corpus sets. Qualitative responses indicated support for online instruction, albeit complicated by topic modeling concerns with the modality. Some twitter responses criticized government actions as restrictive. The investigator concluded that insulation and threats towards habitualization and institutionalization of newer teaching modalities during covid are rich and sometimes at odds with each other, showing tension at times.

## Introduction

Scientists have identified COVID-19 as a highly infectious disease caused by a SARS-CoV-2 virus; they have observed that it has affected the world population, causing roughly 2.9 million deaths worldwide, and they have recorded it as a significant pandemic [1]. As a result, by March of 2020, many schools across the world closed campuses [2–4]. Schools in almost 14 countries would remain closed for over one year [5]. After that, many schools had mandates to teach online in emergency procedures. What follows is what is understood about the experiences of teachers and students through research performed during COVID-19 distancing measures.

## Literature review

One of the most challenging findings of the urgency of the covid-19 teaching mandates is that it required teachers to examine their practices and deal with challenges head-on and speedily. These findings meant developing new plans and policies to counteract the unpredictable nature of the pandemic’s effects on teaching and learning [6–8]. In some cases and some parts of the globe, the immediacy of dealing with covid and its grip upon the teaching profession amounted to working without clear plans and improvising lessons [9–11]. Rapid professional development ensued in many parts of the world, focusing on training teachers to use technology to bridge the gap in online teaching modalities [12–14].

One of the emerging findings resulting from rapid professional development is the quick adoption of emergency remote learning or the temporary use of an alternate model of teaching delivery due to catastrophic situations [15]. Many countries have turned to this mode of learning, using various kinds of technology to meet the learning needs of students. The results have been mixed, leaning towards challenges.

Difficulties have emerged for teachers in dealing with online technologies and learning management software. Some teachers have been hesitant and have crossed their fingers, hoping that they could implement lessons taught about software [16]. In one study, polls taken by information technology departments concerning teachers’ experiences with massively open online courses (MOOCs), or online teaching, showed that many students did not have experience using either modality of instruction [17]. In another study, roughly 45% of respondents stated that they did not find it comfortable to teach online [18].

Other issues emerged germane to the lack of technological literacy. In many instances, developing countries broadcast lessons over television and used quick response (QR) codes to access textbooks. In some developing countries, states delivered paper-based packets of assignments to students due to a lack of internet access [4, 11, 15]. These delivery modalities meant an issue of access to the curriculum was present, in addition to a digital divide cutting across social and economic classes. Related to this was the premise that students must be located in areas where they could receive internet signals to do schoolwork [8, 19]. These limitations meant that students were left out of the curriculum by mere geographic location unless there were innovative ways to work around the lack of wifi or internet cable. Researchers have noted that in some Palestinian households, multiple children had to wait in line to use a single computer shared by parents who were also working online [20]. These arrangements presented social and familial stresses due to the inadequate technology supply in an alternate educational setting.

Other inequities have surfaced in light of covid-19. Some have argued that without schools in session, some students had nowhere safe to go, and they missed the social and emotional support that face-to-face schooling could provide [21]. Likewise, schooling offers nutritional support for grade school students in many instances, and workarounds had to be considered [11]. In some parts of the world, online interventions did not include support for students with special needs, including deaf, blind, autistic children, and so on [19]. These inequalities amounted to missed opportunities for some parts of the world to provide student wrap-around services. In some cases, these inequalities were accentuated by occurring in developing countries.

Teachers have expressed many different feelings about covid-19. One author has noted that teachers have felt a sense of alienation in teaching online [22]. This feeling of separation can be due to the lack of non-verbal communication. To overcome feelings of isolation, one educational institution initiated small online gatherings to connect faculty with one another, called “morning huddles,” as a way to connect more deeply [23]. Finally, one survey conducted during covid-19 teaching indicated that roughly 88% of teachers kept in touch with colleagues on a semiprofessional level, getting tips, sharing ideas, and collaborating around their work [18]. Each of these examples shows the isolation that teachers reported and how they took steps to overcome those feelings.

Teachers also felt that they needed to get closer to their students and that technology hindered doing so. In a survey administered in Australia, responses indicated that students’ lack of engagement had the most direct effect on instructors’ sense of self-efficacy [24]. Other teachers felt that they had no back and forth with students, effectively leaving the teacher as the only one talking during instruction in some cases [18]. One author suggested that a workaround to connecting with students was to interview students with a keen focus on being authentic [25].

Teachers felt that student performance suffered from online learning. For example, one researcher noted that some students were very motivated to work but looked to teachers as guides in instances where teachers could not help [16]. In other cases, the closure of classrooms and the shift to online learning dropped student motivation levels because of the programmatic changes [26]. In a final example, available materials were not culturally appropriate to the country’s context, and as a result, the materials were distracting to students’ learning [20]. In some cases, students responded to the online shift in teaching modality by not turning in work [4].

### Limitations and gap

The study author has viewed some of the limitations of previous literature as quality within in those articles. This lack of high quality is possible because writers had to recruit teachers living through the pandemic while researchers themselves defined the phenomena of teaching through covid. This activity may have resulted in poor quality of recruitment for studies (viz. convenience sampling) [17, 20]. In addition, some of the articles showcased issues with lack of robust methodology [6, 26], and lack of verification documents (viz. audit trails) [12]. Additionally, other papers were written as conceptual, or descriptive papers, without clear methodologies [2, 7–11, 15, 19, 25]. These missing paper aspects would have strengthened the articles’ claims.

Only a handful of articles have dealt with covid and twitter responses to the pandemic. Of these, most have focused on twitter broadly and as a resource for teachers to teach during covid [27–29]. Other articles have focused on teachers’ twitter professional networks as support enclaves throughout the twitter-sphere, finding that twitter posts containing affect rose during covid [30]. In their study about public opinion about online learning and covid, one author has found public support for continued use of online modalities after covid subsides and students return to the classroom [31]. These narrow article domains present a limitation to the cache of articles presented in the literature review. Further, it represents a gap in what is understood about the general public sentiment and what people think and write about teaching, publicly. This paper fills this gap by investigating what is known about public writing on covid-19 in the twitter-sphere.

### Problem statement

Throughout the literature review several researchers have illustrated the issues of dealing with the immediate fallout of covid teaching restrictions, and many researchers have discussed the experiences of both teachers and students dealing with online instruction. However, researchers do not know (in a systematic and well-defined manner) how the general population has reacted on social media to teaching during the covid pandemic. This unresolved question presents a definitive problem for academics, legislators, and politicians who need to know what the public thinks about the different teaching modalities (viz., teaching online versus teaching in schools) that happened during the covid pandemic. Social media responses can apply pressure to situations where large groups of people hold certain positions on schooling from home, for example. Articles have shown that several debates have happened because of pressure parents have placed on legislators to open schools, which affects public policy [32–34]. Therefore, understanding what social media says about the issue of teaching during covid is of great importance. This paper contributes to understanding what people worldwide have written about covid-19 and teaching modalities, and what these ideas have done to insulate habitualized teaching practices or threaten them. The author was motivated to study this problem after reading about the wide variety of public responses to teaching online while holding the space for teaching in schools during the covid 19 epidemic.

### Research questions

1. Are there statistically significant differences in means between scores on the National Research Council (NRC) sentiment valences for two different foci of twitter entries?
2. What are the main topics listed in the corpora of tweets?
3. What do the twitter entries say about teachers in relation to positive and negative sentiments?

## Theory

**Fig 1:**
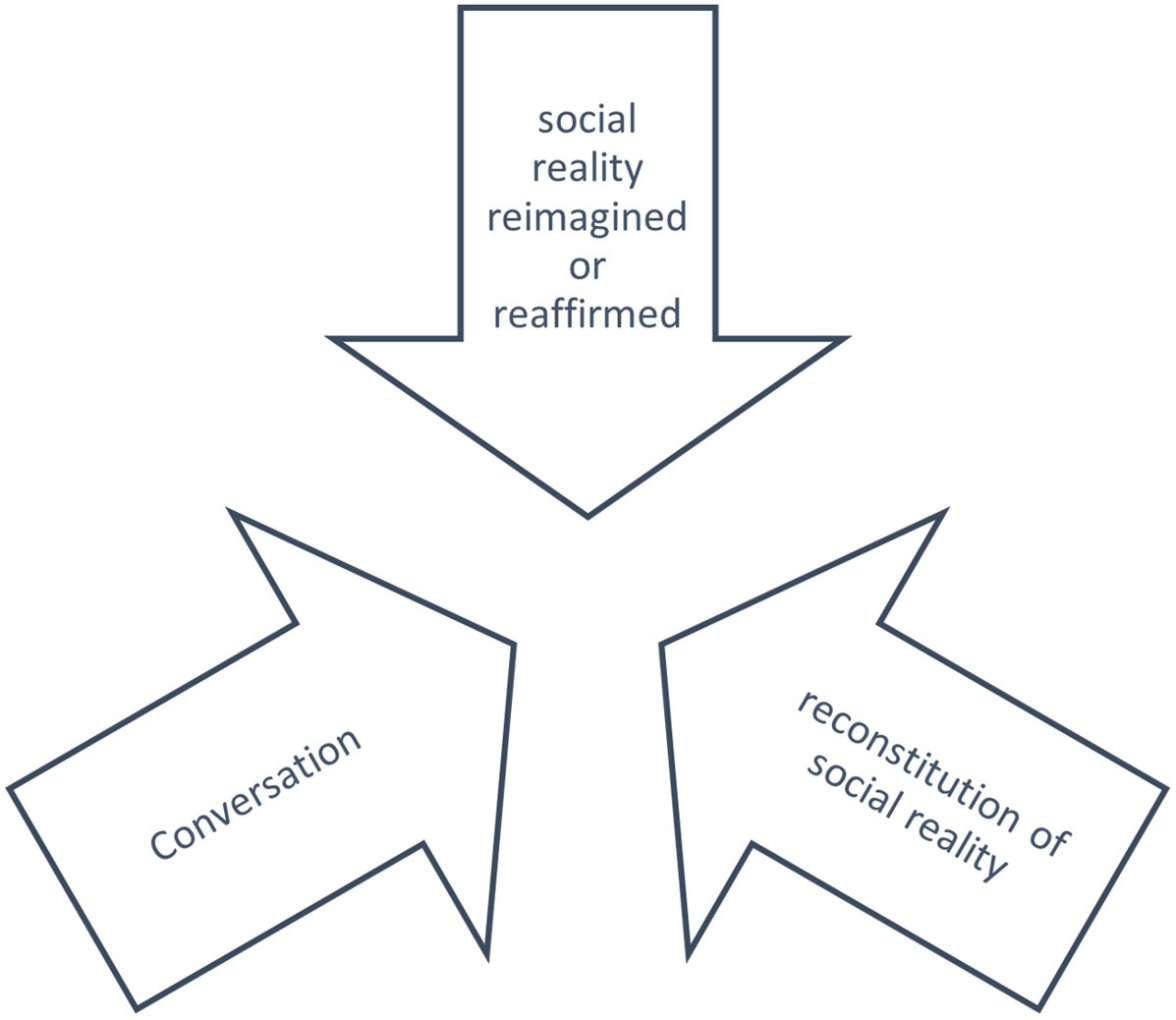
Social construction of knowledge, negotiated, reimagined, reconstituted, in flux.

The theory chosen for the piece is listed in Fig 1 [35]. The study author focused on everyday social life and conversation as a means by which individuals confirm the social world, and reshape and contour it as needed. Here, the mode of discussion is through social media. Theoreticians might say that twitter is the apparatus through which many people might take in ideas and either outrightly reshape the concepts of their social reality or reject these ideas in the sphere of utterances [35]. The result is that several thousands of ideas are in flux at one time, breaking down, reconstituting and reasserting social reality simultaneously (Fig 1).

## Materials and methods

**Fig 2:**
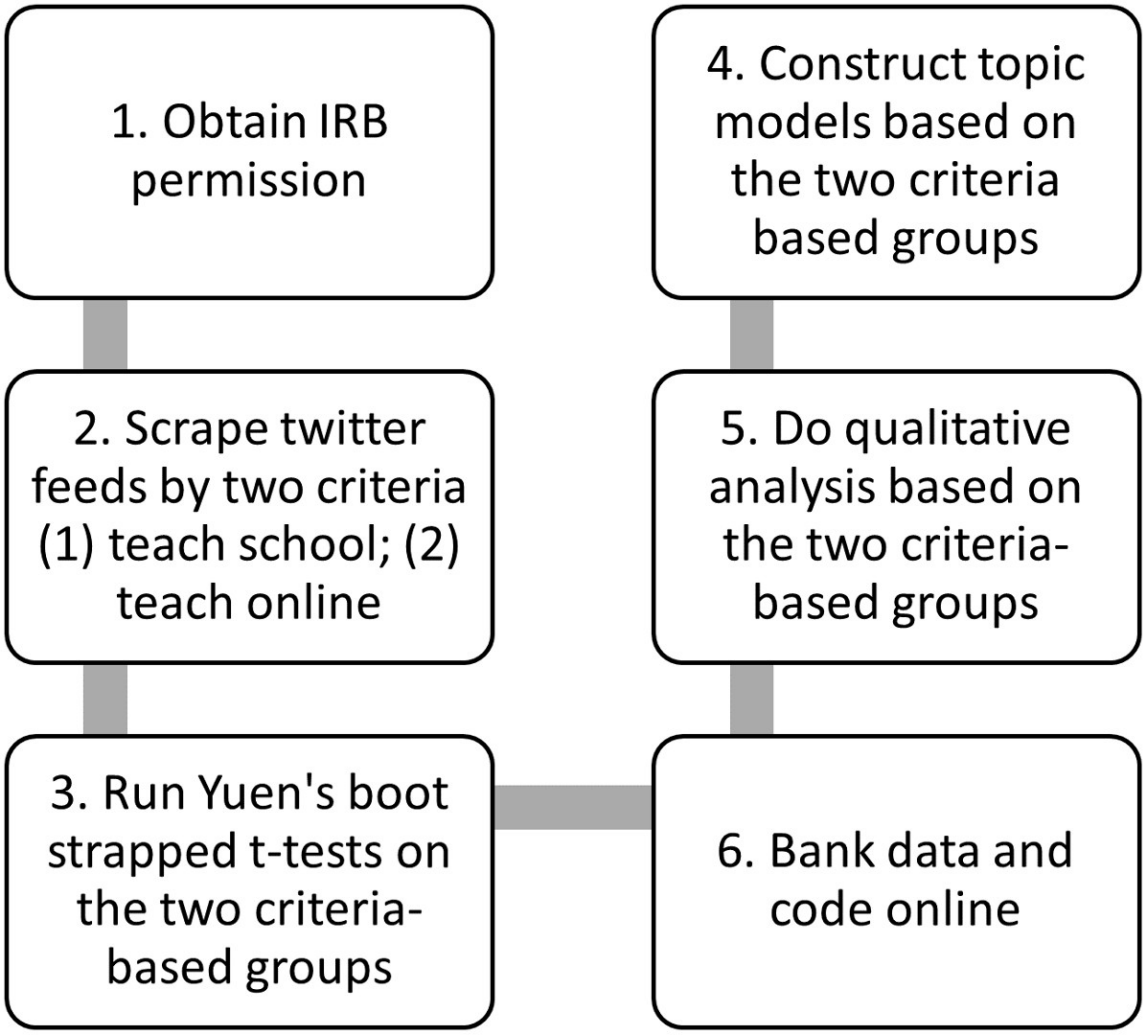
Materials and methods.

Fig 2 shows the materials and methods.

**Fig 3:**
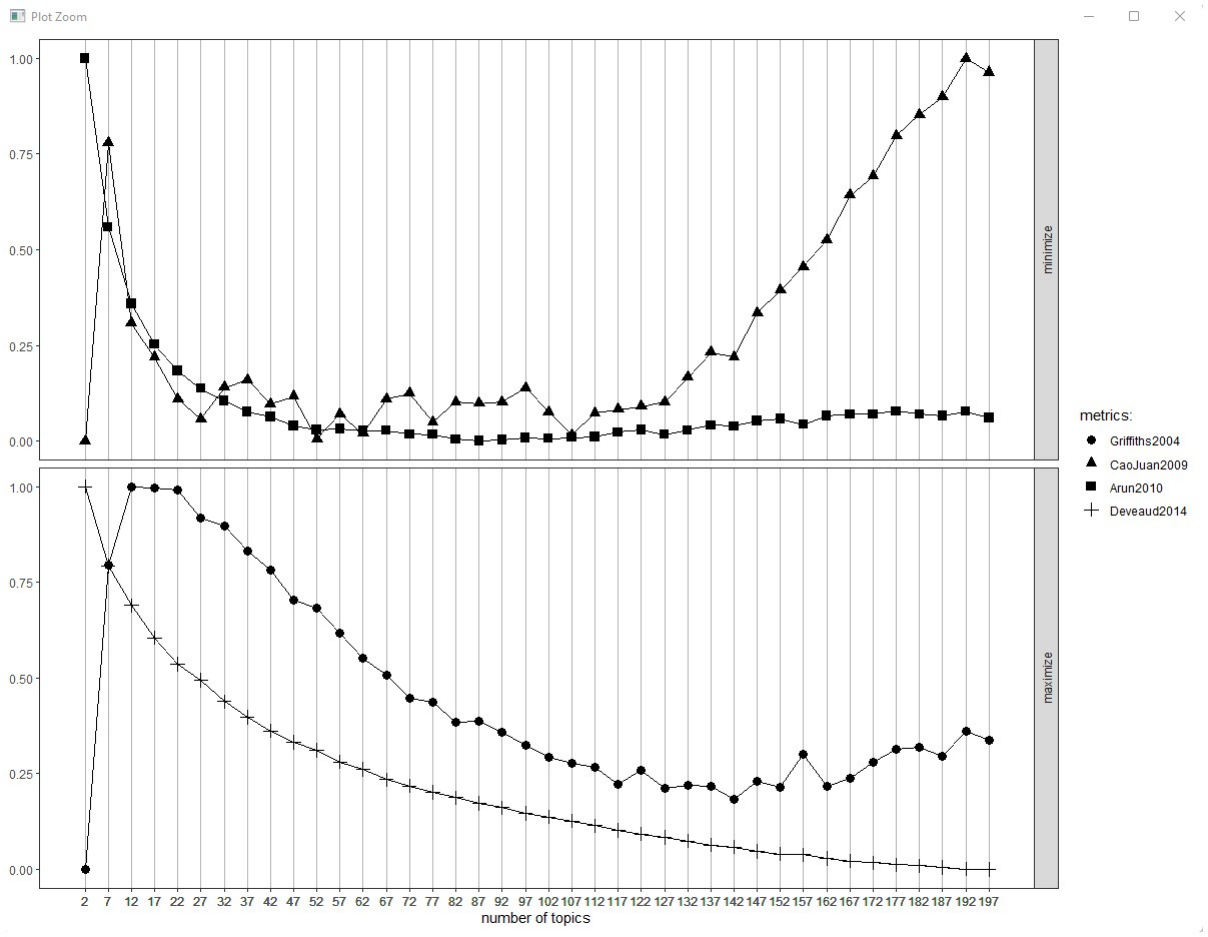
Kappa selection at 57 topics.

**Fig 4:**
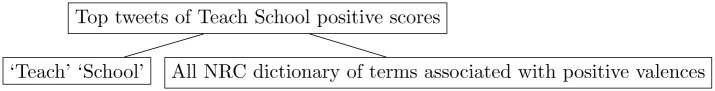
Coding frame 1.

**Fig 5:**
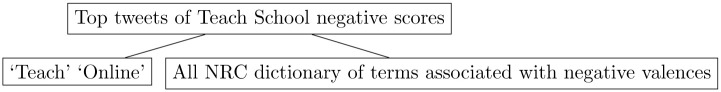
Coding frame 2.

Step 1: Permission was obtained from the Internal Review Board (IRB). The study author submitted the study proposal to The University of Houston, and its institutional review board (IRB) judged it exempt from oversight as the data was publicly available, study #00003019. Tweeters were not required to give informed consent, as the study was not considered research involving human subjects, per the IRB. The study author adhered to the Kozinet’s guide to ethical use of online data in which he advises that investigators protect netography users’ rights by anonymizing data as much as possible and undergoing some kind of ethics audit (which in this case was handled by the IRB review) [36].
Step 2: The data was retrieved using scrape hero cloud, a twitter API [37]. The study author retrieved the data using scrape hero cloud. The date delimiters for collecting tweets were for March 1, 2020 to August 30, 2021. The first group of tweets were retrieved using these search words: ‘covid’, ‘teach’, ‘school’. This yielded n = 8,371 tweets. The second group of tweets were retrieved using the following search words: ‘covid’, ‘teach’, ‘online’. This yielded n = 5,009 tweets. The tweets were anonymized after scraping. The study author then deleted duplicated tweets, and mutually exclusive tweets were retained to the categories ‘teaching online‘, and ‘teaching school‘. After calls for unique tweets between the two sets of data frames were executed, the ‘teach school’ data set yielded n = 7,701 tweets, while the ‘teach online’ data set yielded n = 4,392 tweets.
Step 3: Run Yuen’s Boot strapped t-tests on the two criteria-based groups. The study author used the NRC sentiment analysis instrument to analyze the tweets in each category [38]. This kind of analysis is known as transforming qualitative data in mixed methods studies [39]. These emotional categories include anger, anticipation, fear, disgust, and so on. The instrument was originally designed to test the use of more in-depth emotion-based terms and emotions. The category associations were crowd-sourced and went through further instrumentation and experimentation with subjects to ensure that associations could be used in the lexicon. Once the study author obtained numerical scores for each emotion, the scores were then used to perform Yuen’s robust bootstrapped t-tests [40, 41]. These tests were chosen because the battery of t-tests failed normality tests, and Yuen’s t-test is robust to these kinds of violations by providing trimmed means. These tests compared means between tweeted sentiment scores of the ‘teaching online’ and ‘teaching school’ groups of tweets and produced the t-scores and *p*-values. To understand the high mean scores of the initial quantitative modeling, the investigator created tables to compare the texts of the ‘teaching online’ and the ‘teaching school’ tweets to give sociological perspectives to these glimpses into the larger corpora. The study author then examined extreme cases recorded from the NRC sentiment framework to give insight into the quantitative scores recorded [39].
Step 4: Construct topic models based on the two criteria-based groups. The investigator used topic modeling, which is a form of content analysis [42], to reduce the twitter data into a manageable form; this reduction can be accomplished by indicating word repetition in the body of tweets, which is the basis of the results. The investigator created two separate topic models on the criteria-based groupings to determine the topics contained in the large corpora. This analysis was performed using latent Dirichlet allocation (LDA), an unsupervised mode of classification. Beta scores, or per topic, per word probabilities, were reported [43]. Topic model views can be somewhat subjective, but model tuning can help to reduce subjectivity on views, providing a quasi-subjective framework with which to view the corpus. Model tuning acts like a pair of binoculars, focusing the corpus without under or over focusing word probabilities [44]. Fig 3 shows four model tuning estimators that provide windows into the topic model corpus [45–49]. *κ* = 75 was chosen for both corpora as it gives a view that is consistent with a way of seeing the topics that is not overly repetitive in word probabilities [45]; The study author confirmed the viability of the corpus through human inspection.
Step 5: Perform content analysis based on the two criteria-based groups. As part of a multi-method approach and to triangulate findings [39], the study author took extreme top scores from the NRC valences and performed content analysis on the tweets. The top tweets of the “Teach School” and “Teach Online” negative and positive scores were taken to form the qualitative analysis. These positive and negative themes were chosen to give broad brushstrokes over more nuanced sentiments, while providing general insights into the differences in scores. Two coding frames were set up with the NRC computerized dictionary to differentiate tweets and automatically pull them to form the qualitative analysis (Figs 4 & 5) [50]. Once the tweets were pulled from the corpora, they were thematicized into the qualitative writing. As the investigator worked alone, he left an audit trail for readers to pursue.
Step 6: Bank data and code online. The data and code were banked online for auditing. Information about this part of the methodology can be found in the next section titled, “verification”.

### Verification

In order to provide the essence of reproducible research [51], and therefore, trustworthiness, the study author provided a dynamic document covering the data processing procedurals [51], a link to the raw data for the quantitative analysis which may be found at https://doi.org/10.5281/zenodo.7135938 [52], and a link to the audit for the qualitative data for reproducible code [53], and the positive valences [54], and negative valences referred to in the results section [55]. All data are fully available without restriction.

## Results

**Fig 6:**
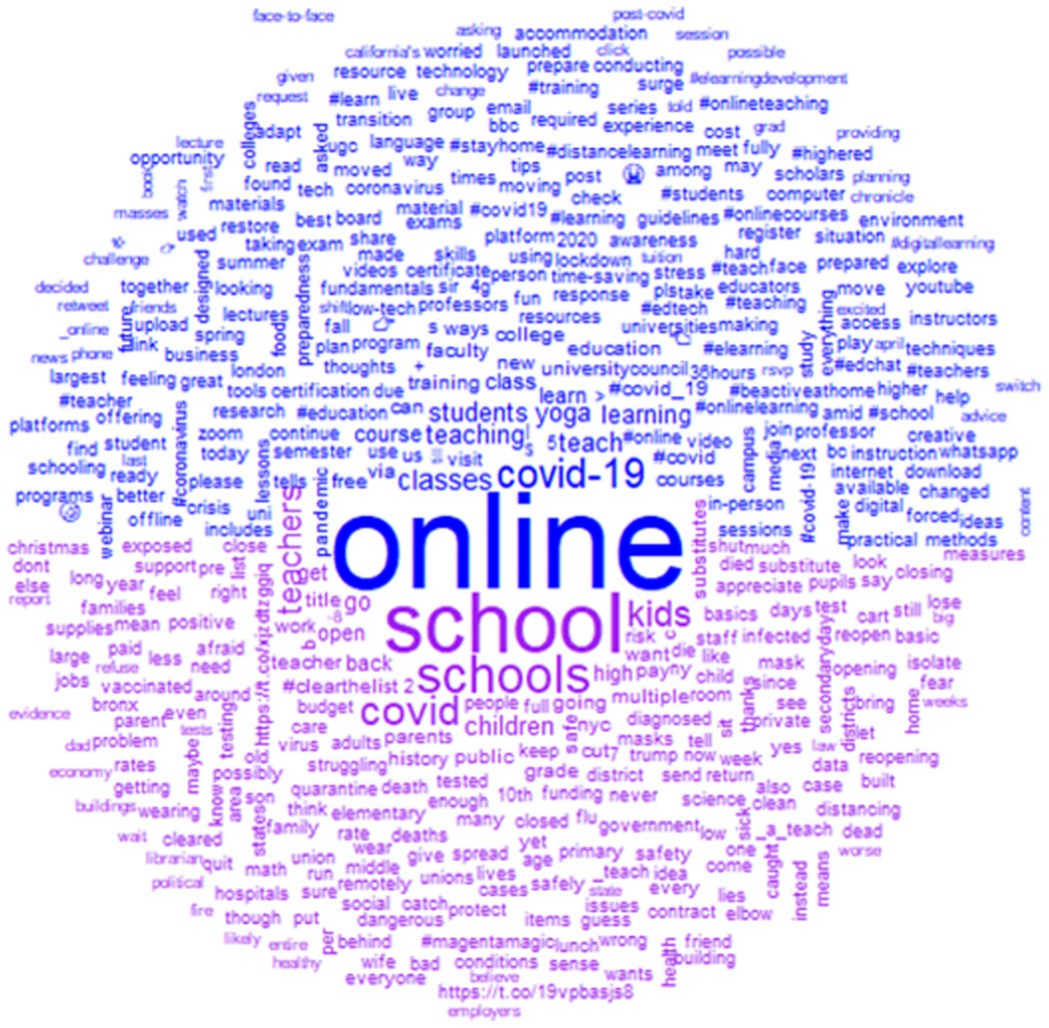
Frequency of terms comparative of ‘teaching online’ and ‘teaching in school’.

It is useful to view the basic document term matrix before sending it off to further processing as it allows glimpses into the corpus. Here the ‘quanteda’ package allows a view into the corpora by splitting the view into frequencies of *n* = 10. Fig 6 shows terms existing into the corpora frequently appearing. This gives us a view that the reader might not otherwise have into all 12,093 tweets at one time, a kind of elevated reading into the document [56]. Corpus 1 is represented by the search terms “teach” and “online”; Corpus 2 is represented by the search terms “teach” and “school.”

### Topic modeling

**Fig 7:**
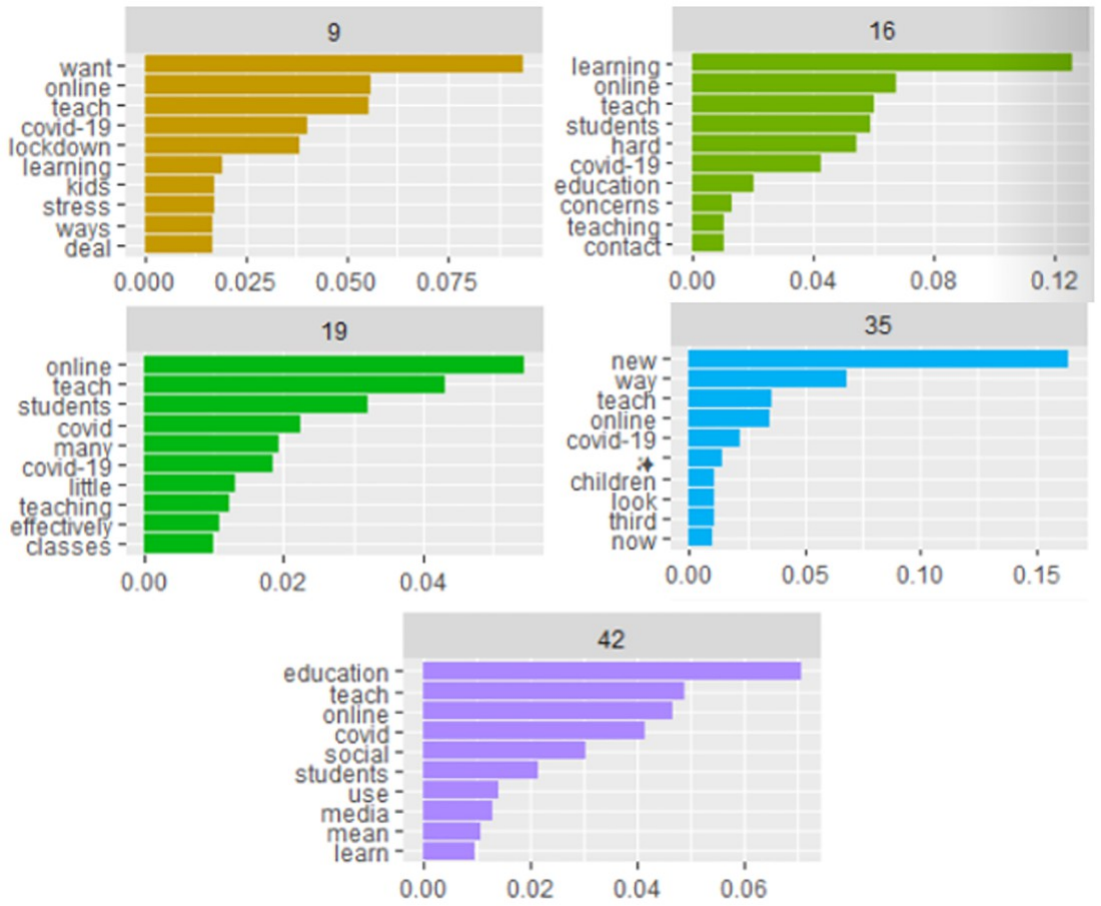
Selected topics topic model 1.

The study author created two separate topic models, one for Topic model 1 (corpus 1) and the other, Topic model 2 (for corpus 2). The first set of topics was produced from topic model 1 (Fig 7). The selection of topics was set at 57. Of these 57 topics, the study author selected five topics with the most pertinent meaning for this exploration. Topic 9 might be interpreted as discussing stress related to online teaching, as the term “stress” appears with beta approximately = .015 in relation to “online,” “teach,” both with betas approximately = .06 and “covid-19,” and “lockdown,” each with betas approximately = .035. Topic 16 might be interpreted as capturing the difficulties of learning online as teachers are not allowed contact, raises concerns, and is hard to do. The study author constructed this interpretation from key words in context (KWIC) searches into the corpus, and the beta values in topic 16. Beta values include that “contact” is approximately.012, “teach” is approximately.06, “hard” is approximately.05, “online” is approximately.07, “learning” is approximately.135, and “concerns” is approximately.015. Topic 19 might be interpreted as commentary on teaching effectively; namely, the opinions set forth that there is little effective teaching happening. The terms bear this out, along with KWIC searches of the corpus; “little” beta approximately.015, “teaching” beta approximately.014, “effectively” beta approximately.013. Topic 35 might be viewed as online teaching being a new modality of instruction. The terms read as “new” with its beta approximately.17, “way” with its beta approximately.067, “teach”, and “online” with their betas both approximately.035. Finally, topic 42 could be interpreted as for some of the subjects students use media to learn. From the topics it might be the topic of social studies, although this cannot be definitively ruled in. This interpretation is derived from the terms: “social” has a beta of approximately.03, “media” has a beta of approximately.014, and “learn” has a beta of approximately.013. Each of these topics give a window into the larger corpus of 4,392 tweets about the subjects pulled up with call terms “teaching” and “online.”

**Fig 8:**
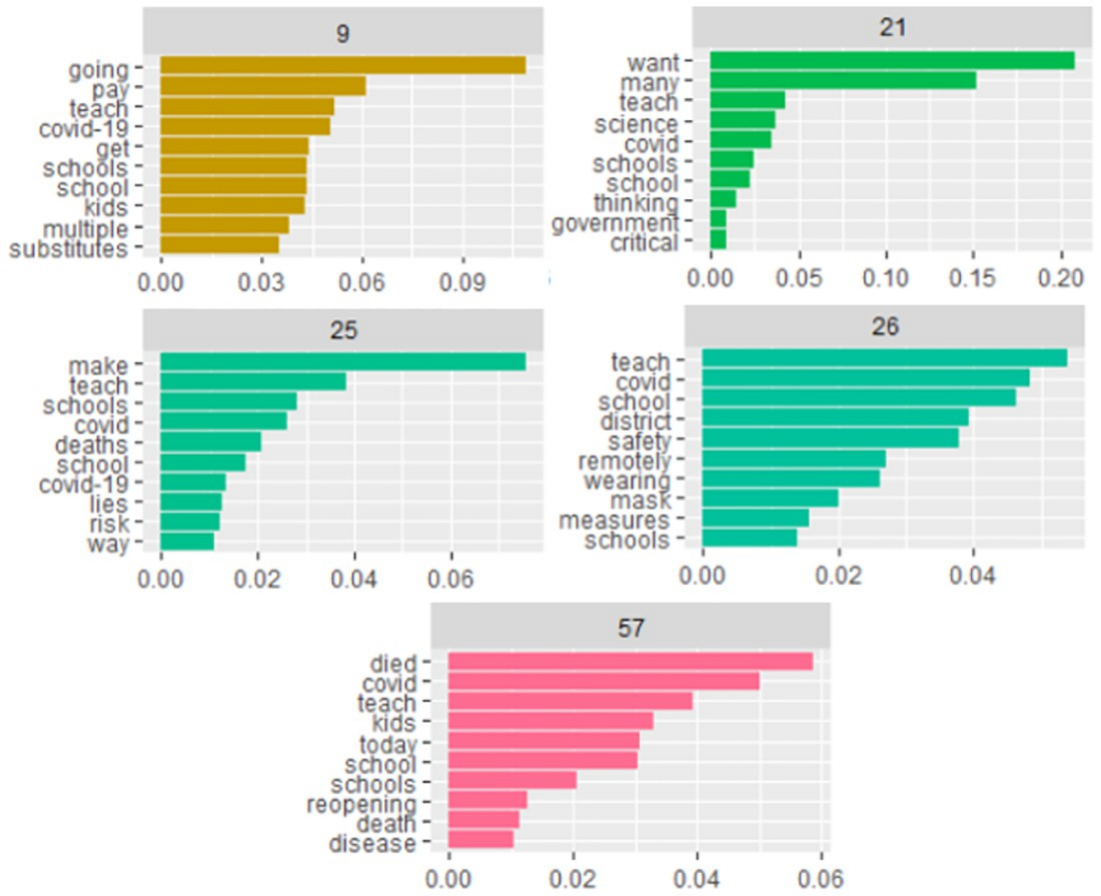
Selected topics from topic model 2.

The study author produced the second set of topics from topic model 2 (Fig 8). Of the 57 topics delineated, five topics were again selected with the most pertinent meaning for this study. Topic 9 could be interpreted as the need for substitutes in the age of covid-19 in schools and the issues it brings up. The beta values bear this out, with “substitutes” having a beta value of approximately.032, the term “pay” with a beta of approximately.06, the term “multiple” with a beta of approximately.035. Topic 21 might be seen as the intersection of arguments between science and schools and government thinking. Again, the terms and beta values bear this out with “science” having a beta of approximately.043, “schools” having a beta of approximately.041, “thinking” having a beta of approximately.03, “government” having a beta of approximately.02, and “critical” having a beta of.01. Topics 57 and 25 each have a discussion of death associated with teaching and schools. Topic 57 have betas of “died” with a beta of approximately.06, “covid” with a beta of approximately.05, “schools” with a beta of approximately.02, “reopening” with a beta of.006, “death” with a beta of approximately.005, and “disease” with a beta of approximately.004. Topic 25 echoed topic 57, but focused on explanations, with lies about risk, perhaps. Beta values include “deaths” with a beta of approximately.02, “school” with a beta of approximately.008, “covid-19” with a beta of approximately.007, “lies” with a beta of approximately.006, and “risk” with a beta of approximately.005. Figure 26 discusses the debate on working remotely versus mask-wearing in schools. The betas showcase this complex discussion. The term “safety” has a beta of approximately.038, while the term “remotely” has a beta of roughly.027. The term “wearing” has a beta of approximately.026, and the term “mask” has a beta of approximately.02. The term “measures” has a beta of approximately.007, and the term “schools” has a beta of approximately.007.

### Quantitative analysis

The investigator ran Yuen’s robust bootstrapped t-tests using the R programming language to test the mean differences in the categories generated by the NRC sentiment lexicon. These results come directly from the measurements on the tweets themselves. The study author made a battery of sentiment comparisons to explore the differences between corpus 1 and corpus 2 between emotional sentiments contained between the two corpora. The results are shown in Table 1. Bonferroni p-value adjustments are included in the table.

**Table 1:** Yuen t-test omnibus tests with Bonferonni adjustments.

Sentiment	*M*_*TS*_	*M*_*TS*_ *SE*	*M*_*TO*_	*M*_*TO*_ *SE*	*t*	Yuen diff	*p*	BF *p*	*d*
Positive	2.76	.002	2.89	.20	4.95	.13	.000	.000	.10
Anger	.39	.01	.26	.01	-11.38	.15	.000	.000	.22
Anticipation	.79	.01	.84	.01	2.84	.06	.003	.027	.06
Disgust	.31	.01	.18	.01	-10.16	.08	.000	.000	.15
Fear	.71	.01	.49	.01	-12.70	.20	.000	.000	.25
Joy	1.53	.01	1.51	.01	-2.33	.04	.020	.180	.04
Surprise	1.27	.01	1.25	.01	-3.24	.04	.008	.072	.06
Negative	1.18	.01	.82	.01	-17.11	.43	.000	.000	.34

Table 1 reports means, standard errors, t-values, the Yuen’s differences (subtractions of the higher mean from the lower mean), *p*-values, Bonferonni corrected *p*-values, and Cohen’s robust delta (*d*) as an effect size. The study author set the mean trim level at 0.2, and the number of bootstrap samples was set at n = 599, the default values for the R function.

Table 1 shows that for only two emotions, tweets about online modalities scored higher than tweets about teaching in schools. Tweets about teaching online scored slightly higher in positive sentiment (*M* = 2.89, *SE* = .20) than tweets about teaching in schools (*M* = 2.76, *SE* = .002), *t* = 4.95, *p* = .000, *d* = .10 (Note: Yuen t calculations do not produce degrees of freedom calculations when bootstrapping). The differences in means were small, and the effect sizes were small. Tweets about teaching online scored higher in anticipation (*M* = .84, *SE* = .01) than did tweets about teaching in schools (*M* = .79, *SE* = .01), *t* = 2.84, *p* = .003, *d* = .06, with differences in means being small, and effect sizes were small.

However, Table 1 showed statistically significant differences between the two groups, with larger differences with more negative emotions. When it came to anger, tweets about teaching in schools scored higher (*M* = .39, *SE* = .01) than tweets about teaching online (*M* = .26, *SE* = .01), *t* = -11.38, *p* = .000, *d* = .22. The Yuen difference between means was.15, with a small to medium effect size. Tweets about teaching in schools scored higher in disgust (*M* = .31, *SE* = .01) than did tweets about teaching online (*M* = .18, *SE*.01), *t* = -10.16, *p* = .000, *d* = .15. this represents a Yuen’s difference of.08, and a small effect size. When it came to fear, teaching in schools tweets scored higher (*M* = .71, *SE* = .01) than did teaching online tweets (*M* = .49, *SE* = .01), *t* = -12.70, *p* = .000, *d* = .25. When it came to negative emotions, tweets about teaching in schools scored higher (*M* = 1.18, *SE* = .01) than did tweets about teaching online (*M* = .82, *SE* = .01), *t* = -17.11, *p* = .000, *d* = .34.

Table 1 also showed a few emotional valences that were not statistically significant. Tweets about teaching in schools focused on joy (*M* = 1.53, *SE* = .01) were not statistically significant from tweets about teaching online focusing on joy (*M* = 1.51, *SE* = .01), *t* = -2.33, *p* = .1, *d* = .04. Finally, when it came to surprise, tweets about teaching in schools (*M* = 1.27, *SE* = .01) were not statistically significant in comparison to tweets about teaching online (*M* = 1.25, *SE*.01), *t* = -3.24, *p* = .07, *d* = .06.

### Qualitative analysis

The top tweets of the “Teach School” and “Teach Online” negative and positive scores were taken to form the qualitative analysis. The results are segregated into paragraphs in this section of the paper. This section is set up as a comparison section between online and school reportings. The analysis in this section starts with positive reportings on tweets. Citations are used to reference the audit which is cited earlier in the document for this portion of the qualitative findings.

Some twitter articles report on the overall model tunings and findings on large machine learning models. These have showcased the power of big data analytics [57–61]. Still other articles report on the health findings about masking, vaccines, and overall health discourse [62–64]. These findings complement the overall findings in the present article, but they mostly add context to the writings that are found specifically about teaching and the modes of education being either online or in schools. Therefore, they set the stage of the close analysis that follows.

Many online tweets expressed thanks for the teachers that were willing to teach under the circumstances. For example, one tweet mentioned teacher appreciation week, and “thank[ing] all the amazing, dedicated teachers in the Berks County!” (T1A4) While this tweet may have been seen as a regularly occurring holiday, it went on to “Thank you for everything you do, including working so hard to develop new & creative ways to teach students online during this COVID-19 health crisis.” (T1B4). Another tweet highlighted that “Musical School + Yumu,” was, “shortlisted for a Teach Primary Award,” because it, “supported 3/4m + children learning & making music during COVID.” (T1A6). In yet another cause for positive reporting, another tweet stated, “Our students, faculty and staff have been amazingly adaptable and creative with the changes we’ve made in response to COVID-19.” (T1A9). Finally, a tweet mentioned that, “Award-winning faculty at Appalachian State share how they keep students feeling connected and supported as they teach online during the COVID-19 situation.” (T1A14) This tweet was followed by links. Other tweets focused on the gratitude felt over being able to teach at a distance. One tweeter put it: “Finally, today, I received a medical accommodation to teach all my classes online this semester. My diabetes puts me at high risk of complications from COVID-19, and I am grateful for the opportunity to try to stay safe this semester” (T1A43). These tweets echo some of the tweets found in other literature that mention joyful statements which encourage doctors in their fight against covid [57], but these remarks are very rare in the literature of twitter findings overall.

In comparison, school tweets focused on a fear of returning to in-person teaching, even though there were positive twists in some of the tweets. For example, one tweet commented that, “For context, wife is a K-5 gym teacher; sister is registrar, and step-dad is professor and department head at university; aunt and uncle both elementary principals, dad, trade school instructor, grandmother HS French and librarian…these share SAME desire to teach but covid fear” (T1B7). Another tweet noted that, “The mental scars from losing a loved one important to them because of covid FAR out weigh the scars of missing school. There are so many solo avenues of learning right now these littles can benefit from. Learning is constant for them. You become the teacher. So TEACH. AND ENJOY.” (T1B8). Each of these tweets show a mixed response of negative and positive text that registered on the lexicon. This dual emotion of fear mixed with positive feelings was documented in the literature, where it was noted that fear was represented in twitter covid corpora that simultaneously in some cases also held, although these latter documentations were more about vaccine availability in the UK and more general sentiments, respectively [57, 58, 62].

Online tweets expressed complaints generally; these surfaced as frustrations over face to face as opposed to online modalities of teaching. One tweet noted, “I am mad as hell that we are basically being for[c]ed to immerse ourselves in a pitri-dish ripe with opportunity for the Covid virus to flourish and hurt kids and possibly kill teachers. The data shows we should teach online.” (T2A1) This tweet was posed on July 25, 2020, relatively early in the pandemic. In another example, another tweet read, “My (online learning) son’s elementary teacher is taking a medical leave because she feels unsafe having to teach in person with a medical condition (hybrid model). And the first COVID case popped up in their school. I have a bad feeling things are about to fall apart here” (T2A2). In some cases early in the pandemic complaints were lodged that people were requested to teach face to face and online teaching was the exception to the rule: “My…state university is now requiring people who have requested to teach online in Fall 2020 due to the ongoing COVID-19 pandemic to provide doctor notes. Is this egregious mistrust and/or common practice? Are others experiencing this?” (T2A10). Finally, some instructors were willing to sacrifice going back to face to face instruction in the early part of the pandemic to enhance instruction. One tweet read, “I’m a teacher who is a diabetic and at high risk of dying from CoVid-19. I’m ready to go back because kids need the interaction and face to face instruction. Data is overwhelming that online doesn’t teach kids. My students have higher risk of dying from the flu statistically.” (T2A15). In each of these tweets, frustrations emerge in some way regarding face to face teaching, whether online teaching predominates, or whether online teaching has yet to become the norm. The idea of complaints has been noted as something that could be learned about during the crisis and was a focal point for some researchers alongside what could be appreciated during the covid pandemic [63].

In some cases, online tweets showed that complaints were lodged at leadership in online teaching. In one quote, the speaker stated, “[The government] is winging it rather than planning. Dreadful leadership expecting staff to teach face to face and online, track and trace, fund ppe and sanitiser, test for COVID, refuse schools to close, oh and delay payments and no pay rise. All at the last minute.” (T2A9). Another tweet read, “[T]here is no doubt that the Covid infection will increase drastically effecting everyone who comes to the college. As per government order, online/offline/integrated media is used to teach students. If this is the case, the tests should also be online/offline.” (T2a15). A final tweet noted that, “The university sector is being left to deal with the problem of Covid on its own; the government is showing a lack of leadership. It is possible to teach online. Face to face teaching is a risk to students, staff and local communities.” (T2A23) Trust in government was found to be a theme in other studies, particularly when it came to covid vaccine acceptance [62]. However, there were negative responses in the literature that captured overall disappointment on national responses to the pandemic that echos the sentiment captured here [65].

School tweets had a focus on students falling behind in some cases. For example, “Does the Covid allowance take into consideration teachers who need to isolate? And are unable to teach face to face. Schools are under a huge amount of pressure to get sub teachers that are subject specific, due to the high absence. Students are losing out on valuable learning[.]” (T2B30). In another example, a tweet read, “[The American Academy of Pediatrics] calls for students to return to school. So torn given how hard it is to remote learn/teach, lack of social skills, falling behind in class and safety of staff/kids.” (T2B31). These two examples showcase how in the negative school tweets there was a focus on students falling behind in their work due to covid-19.

Other focal points in the findings reveal an emphasis on instructors surviving or having to live through covid. One tweet stated, “My sister who was forced to teach in person is now Covid+, so is her daughter now. You did this to her. Close k-12 schools.” (T2B11) In another tweet, the writer noted, “If hospitals are overwhelmed, people with emergency conditions other than Covid are shut-out as well, increasing mortality…If so many are sick/dying, who will teach schools, run stores, distribute food? Not sure what it will take for folks to wake-up.” (T2B14). In another example, a person wrote, “…Private school educator from the Southside here. I caught COVID…at school…and now I can’t smell…many months after infect. Slowly getting reconciled to this loss. Still, I can teach in person. I don’t fault my many students/parents who are wary. This situation is complicated.” (T2B21). In yet another example, a tweet read, “…What about the teachers who may not be resilient to the virus? Its bad when teachers have to call out sick bc of a cold. What happens when they have covid, and can’t teach?” (T2B23). These responses resonate with general twitter responses found in studies that indicate overall emotions such as sadness, fear, anticipation and a spectrum of other feelings [59].

## Discussion

Earlier in the literature review it was established that covid-19 represented a sea change in instructional modalities [12, 13, 15]. Early literature reported on habitualizations of ideas, where ideas were just becoming standardized into practices, that then turned into institutionalization through policy moves [66, 67]. These events and movements concretized the social order of things, and have started to become a part of secondary socialization for new and experienced teachers entering and working in the field during the covid-19 era. Therefore, it is fair to say that instructional modalities have changed substantially during March 2020 to August 2021.

However, it can be argued that social media, especially tweeting, is having a conversation, a protracted set of declaratives or interrogatives between tweeters. These tweets can be captured for their descriptions, reactions, and emotional valences towards teaching in response to the institutionalization of the new teaching toggling between teaching in school and online during the time captured in this article. Collectively the tweets show themes that have the potential to reimagine or reaffirm social reality for thinkers during the time of sea change and beyond. It is this tug between challenging and reaffirming that has been explored.

Qualitatively, the positive valences might be interpreted as a way to reinforce the prevailing habitualization and institutionalization of practices of teaching online. Tweets that recognized innovative practices during the pandemic indicate that institutionalization between March 2020 and April 2021 was not closed off, but rather evolving into habitualized or in the very least, emerging practices. This is because various technologies such as vaccines and masks made dealing with covid-19 a moving target, with various responses showing this. The literature has shown that habitualization can be the very beginning of unconscious practices that workers perform without thinking, and in repetitive fashion. This has been shown in other forms of labor such as in work with information systems, for example [68].

Negative valences served to challenge the institutionalization of teaching and schools although they showed up in tweets categorized in teaching and online. This is owing to the fact that pattern matching commands searched for teaching and online, but the conversation, although leading into teaching and online subject matter, was going from teaching about schools, although being mutually exclusive from the pattern matching that categorized the latter. The study of institutionalization of online learning has occurred in higher education since at least the early 2000s, and has found that some universities embrace managerial practices of habitualization over more scholarly approaches to management faculty [69], leading to institutionalization of online learning [70]. This contrasts greatly with what has typically happened with k-12 schools, which have traditionally gone face to face in teaching modalities. Under the struggle shown in this study, tweeters have challenged the institutionalization of online learning.

Some of the negative valences critiqued leadership and policy that early on wanted online teaching as exceptions. This again shows that the role of covid-19 and school policy was a moving target from March 2020 to April 2021. Leadership was a target noted during the changes, as well as government agencies in some countries that were responsible for handing down public policy. Some tweets discussed neglect and others focused on restrictions imposed by the government. Finally, other tweets focused on the government taking a hands off approach entirely. This is a bottom up critique of habitualization and institutionalization of public policy, as it has its reach far up beyond the local level, finding its grip in the reach of elected officials, especially. However, as a crisis response, leadership had to implement online learning and respond with crisis leadership approaches, which included adaptation to the pandemic [71].

The topic modeling indicates that the corpora was focused on particular themes. Among them in topic model 1, where a focus is placed on teaching online as a new modality. This is challenged by topic 19, where the betas lead an interpretation that might read that little effective online teaching is taking place. Topic 16 echoes topic 19, which might be interpreted as inferring that online teaching is difficult and that there existed concerns over the process. It could be inferred that there is a weak insulating force that is simultaneously threatened by complaints against newer forms of habitualization of teaching practices.

In contrast, topic model 2 shows that teaching and schools conjures up its own issues. One of the issues is the problem of substitute teachers, as seen in topic 9. Another more serious issue is the glaring problem of potential deaths associated with teaching in schools, captured by both topics 57 and 25. The lack of substitute teachers was an issue marked in other forms of social media at the time, and has been noted during the pandemic [72]. Finally, topic 21 reveals the problem of wanting an association of critical thinking about science with the government about covid and schools, it might be inferred. Both sets of topics show radically different sets of concerns and issues related to covid and teaching. From here it might be interpreted that older institutionalized forms of instructional modalities are threatened by the twitter sphere. Here, this means that teaching face to face became solidified as instructional types that receive little support in talk.

## Conclusions

There are several major conclusions drawn from the study. It is concluded that covid 19 represents a sea change for teaching modalities across the world. Habitualized practices such as teaching face-to-face that been institutionalized for so many years had been challenged in the wake of the virus. The virus’ public policy precautions has caused so much backlash that they have in some cases threatened those policies put in place to slow the spread of the virus in schools. This study showed that teaching online tweets showed less fear, anger, disgust, and negative emotions than did teaching and school tweets. If triangulating with the other evidence in the data, it might be due to there being less preoccupation with death, fear of disease, and risk. Online teaching comments served as insulating forces, supporting efforts at providing resources for teachers to provide online lessons to students. However, it was noted that not all of these efforts were successful. Some of these efforts were viewed as difficult and led to perceived concerns. Teaching face-to-face comments included critiques towards governments, particularly for their perceived neglect of the situation, presupposed laissez faire attitude, and subjectively viewed restrictions imposed on the public. Finally, it is concluded that the push and pull of insulation and threats to habitualization are rich and sometimes at odds with each other, showing tension at times.

### Future research directions

There is no doubt that covid-19 has changed the way schooling continues to transpire throughout the world. It has introduced new practices and technologies into the classroom. Several research questions follow. For example, what newer technologies have persisted in the post-covid teaching environment? How have those technologies been modified to stay relevant? Now that teachers have had experience with newer forms of technology, what do they perceive are their strengths and weaknesses? Which students succeeded the most in online learning, and how can more students succeed in online learning as we advance? The answers to these questions may impact the use of educational technology as time goes on.
